# Posttraumatic Stress Disorder, Depression, and Prolonged Grief Disorder in Families Bereaved by a Traumatic Workplace Death: The Need for Satisfactory Information and Support

**DOI:** 10.3389/fpsyt.2019.00609

**Published:** 2019-08-30

**Authors:** Lynda R. Matthews, Michael G. Quinlan, Philip Bohle

**Affiliations:** ^1^Work and Health Research Team, Faculty of Health Sciences, The University of Sydney, Sydney, NSW, Australia; ^2^School of Management, UNSW Business School, University of New South Wales, Sydney, NSW, Australia

**Keywords:** traumatic bereavement, industrial deaths, information, support, mental health, PTSD, family, injury fatality

## Abstract

The impact of traumatic workplace death on bereaved families, including their mental health and well-being, has rarely been systematically examined. This study aimed to document the rates and key correlates of probable posttraumatic stress disorder (PTSD), major depressive disorder (MDD), and prolonged grief disorder (PGD) in family members following a workplace injury fatality. The hidden nature of the target population necessitated outreach recruitment techniques, including the use of social media, newspaper articles, radio interviews, and contact with major family support organizations. Data were collected using a cross-sectional design and international online survey. The PCL-C (PTSD), the PHQ-8 (MDD), and PG-13 (PGD) were used to measure mental health disorders. All are well-established self-report measures with strong psychometric qualities. Participants were from Australia (62%), Canada (17%), the USA (16%), and the UK (5%). The majority were females (89.9%), reflecting the gender distribution of traumatic workplace deaths (over 90% of fatalities are male). Most were partners/spouses (38.5%) or parents (35%) and over half (64%) were next of kin to the deceased worker. Most deaths occurred in the industries that regularly account for more than 70 percent of all industrial deaths—construction, manufacturing, transport, and agriculture forestry and fishing. At a mean of 6.40 years (SD = 5.78) post-death, 61 percent of participants had probable PTSD, 44 percent had probable MDD, and 43 percent had probable PGD. Logistic regressions indicated that a longer time since the death reduced the risk of having each disorder. Being next of kin and having a self-reported mental health history increased the risk of having MDD. Of the related information and support variables, having satisfactory support from family, support from a person to help navigate the post-death formalities, and satisfactory information about the death were associated with a decreased risk of probable PTSD, MDD, and PGD, respectively. The findings highlight the potential magnitude of the problem and the need for satisfactory information and support for bereaved families.

## Introduction

Global trends released annually by the International Labor Organization reveal work is a significant source of death and bereavement. Every day, approximately 6,400 people die from work-related causes (1), and each year, 300,000–400,000 people die from traumatic work injuries (2). However, these estimates provide a “false picture of the scope of the problem” due to the extensive underreporting of fatal injuries and diseases (3). The majority of all workplace deaths occur in low- and middle-income countries where occupational health and safety (OHS) is often not even recognized as a public health priority (4), and OHS-related conventions have yet to be adopted (5). Unfortunately, workplace deaths continue to occur in rich countries with longstanding regulatory prevention. In 2014, there were 188 fatal traumatic injuries at work in Australia, 919 in Canada, and 4,821 in the United States. Each death potentially affects up to 20 family members and close friends (6). Despite its prevalence, the impact of traumatic workplace death on families remains largely unexplored.

Although there is a growing body of research on the psychological impact of death on families and communities—including the death of children, suicides, crimes of violence, accidents, and drug and disease-related deaths—the consequences of fatal work injuries for families are relatively unknown (7). The few studies that have examined death and bereavement in the workplace have seldom considered workers’ families (8–10). The same can be said of large workplace disasters, such as the explosion on the Piper Alpha Rig in 1988 (11), which killed 165 of the 226 men on the offshore rig and, more recently, the Pike River Coal Mine disaster where 29 miners died (12). These disasters have been examined in detail by researchers, but the impact on next of kin and families has not been investigated. Given that previous research into traumatic deaths arising from terrorism and natural disasters has found significant psychosocial effects on communities (13–15), the lack of a similar body of research on the impact of industrial deaths on families is arguably a significant omission.

Broader evidence indicates that people confronted with the traumatic and violent death of a relative have increased risks of PTSD, major depressive disorder (MDD), and prolonged grief disorder (PGD) (16–18). For example, family members bereaved by homicide have rates of up to 34 percent for PTSD, 54 percent for depression, and 44 percent for PGD (19–21). Although broader evidence on responses to traumatic deaths provides some guidance regarding families’ experiences following a sudden workplace death, distinctive characteristics, particularly in terms of regulatory responses, mean that they are not directly comparable to sudden deaths from crimes of violence, suicide, or road trauma.

Sudden workplace deaths involve specific law dealing with OHS with its own requirements, inspectorate, and prosecutorial regime and dedicated workers’ compensation laws. Extensive critique of the OHS legislation suggests that it is unable to adequately enforce regulatory laws, that breaches are under-enforced, and “many culpable failures to control health and safety go unpunished” (22). OHS offences are not considered to be “really criminal” (23), prosecution is a “last resort,” and the consequences of prosecutions are usually inconsequential (24, 25). Further, the response involves an array of institutions including employers, specialist insurers, specialist law firms, unions, and (sometimes) victim advocacy bodies, as well as coronial courts—systems that families need to navigate during their bereavement (26–28). This institutional and regulatory apparatus is highly developed in almost all high-income countries (the major exception being those in the oil gulf states) (29), but less so in poor- and middle-income countries that have under-developed and more lopsided regulatory apparatus. Although these factors have been little researched, a study of Chinese seafarers identified the grief experienced by families which was exacerbated by their inability to obtain workers’ compensation in many instances (let alone prosecution of culpable parties) with Communist Party-controlled unions failing to really represent compensation claims (30).

Given this context, it is possible that the impact of sudden workplace deaths on families is exacerbated because their loved ones died in working environments in which safety is apparently heavily regulated by OHS law, and workers should be protected from catastrophic injury. Except for some vocations like emergency services and the armed forces, there is a strong community expectation that going to work should not entail a threat to life. This expectation has been the specific focus of media campaigns on workplace safety run by regulatory agencies, typified by scenes where a child waits at a gate for their parent to return [for example, Ref. (31)]. The scarcity of research in this area means that little is known about the predictors of PTSD, MDD, and PGD among those whose relatives are fatally injured at work. The evidence from studies on the consequences of sudden and violent deaths indicates that levels of psychopathology are higher among women, those with a psychiatric history, the more recently bereaved, those who have a close kinship to the deceased, and those who experience the death of a younger relative (32). For example, PTSD is more likely to develop in bereaved spouses when the cause of death is from unnatural causes, such as suicide or accidents, and it almost always co-exists with depression (33). PGD is more likely to develop when the death is relatively recent and due to violent causes (34), and it coexists with depression in up to 50–70 percent of people with PGD (35). Do these factors similarly increase risks for people bereaved by a workplace death? A lack of solid evidence about predictors of these MHCs severely limits the development of interventions to minimize their impact.

Providing satisfactory information and support following a traumatic bereavement is central to alleviating distress and facilitating adaptation (36–39). In particular, satisfaction with social support has been associated with lower levels of PTSD, complicated grief, and depression (40–42). Following a workplace death, information and support may be provided to families by authorities involved in regulatory responses, such as the government safety authority or the coroner’s court. Early qualitative research based on interviews with a small sample of family members emphasized the importance of keeping families informed of progress with regard to investigations and importance of flexibility in the timing and provision of support (24, 43).

Survivors of suicide have reported that one of the most needed types of help was specific information about their relative’s death (44). There is also evidence that difficulty accessing information following homicides may increase the chances of family members developing MHCs (21, 45). However, a search of the existing literature found no published studies that explored whether information and support provided by authorities following a workplace death can reduce the risk of family members developing PTSD, MDD, or PGD. The lack of research on this issue means that the nature of the psychological impact of the death on families is unclear, and the adequacy of the information and support provided after the death in reducing the psychological impact is unknown.

Given the lack of evidence on the psychological impact of workplace deaths on families, and the role of demographic variables, information, and support in the development of MHCs, this study has three aims. The first is to collect evidence about the rates of PTSD, MDD, and PGD reported by families bereaved by a fatal work injury. The second is to identify demographic, information, and support variables that are significantly associated with each MHC. The third aim is to identify the variables that are significantly associated with a decreased risk of developing PTSD, MDD, and PGD following the fatality. Drawing on previously identified predictors of PTSD, MDD, and PGD following sudden bereavement, we hypothesize that a shorter time since death, having a mental health history, younger age of the deceased relative, and dissatisfaction with information and support provided following the death will be significantly associated with probable PTSD, MDD, and PGD.

## Methods and Materials

A cross-sectional online survey was conducted as part of a larger, multi-method project that examined the impact of workplace death and institutional responses on surviving families. The criteria for inclusion were (1) being over 18 years of age, (2) ability to read and write in English, and (3) having had a family member die suddenly from an injury sustained from a work process. Fatalities from workplace diseases, such as mesothelioma, or from medical conditions, such as a heart attack or stroke, were excluded.

Being a hidden population, participants were recruited using an advertisement that contained details about the study and the internet address for the study’s home page. The advertisement was distributed to well-established industry and community support networks and promoted *via* social media (Twitter, Facebook), trade union websites, radio interviews, industry newsletters, and newspapers. Interested family members were directed to the study home page where they accessed an ethics approved Participant Information Sheet that provided details about the study. Included in this document was advice that submitting the survey would be treated as informed consent to participate in the study (46). The home page also provided access to the survey link, and the telephone and e-mail details of one of the investigators. This study was carried out in accordance with the recommendations of the National Statement on Ethical Conduct in Human Research (46). All subjects provided freely given informed consent in accordance with the Declaration of Helsinki (47). The protocol was approved by the University of Sydney’s Human Research Ethics Committee (#14981).

### Participants

A total of 207 people, primarily from Australia and North America, accessed the survey between November 2013 and November 2015. Seven (3.4%) did not meet inclusion criteria, six (2.2%) did not provide any data, 26 (12.5%) provided only demographic data, and 20 (9.6%) provided minimal or no mental health data. All these participants were excluded from analyses, resulting in a final sample of 148 participants (71.5%). The 26 participants who provided only demographic data included a significantly larger proportion who were not immediate family (χ2 = 22.836, [*df* = 4], *p* < .001) and were significantly younger (*M* = 42.32, *SD* = 12.35) than those in the final sample (*M* = 48.07, *SD* = 12.43; *t* = −2.171, *df* = 190, *p* = .014). The 20 participants who did not complete the mental health items did not differ significantly on demographic variables from the final sample.

### Measures

#### Mental Health Conditions

PTSD was measured using the PTSD Checklist (PCL) (48). The PCL has been shown to have excellent internal consistency, test–retest reliability, and convergent validity among both clinical and nonclinical populations (49–51). Internal consistency was high in our sample (Cronbach’s α = 0.95). The PCL also has high sensitivity and specificity in relation to the Clinician Administered PTSD Scale (52, 53). In line with previous use with civilian groups, a score of ≥ 44 was used as the cutoff for a probable PTSD diagnosis (52, 54).

The Patient Health Questionnaire-8 (PHQ-8) (55) was used to measure MDD and had high internal consistency (α = 0.94) in our sample. The PHQ-8 has a sensitivity of 88 percent and specificity of 88 percent for a DSM-IV diagnosis of major depression using a cutoff score of ≥ 10 (56). This cutoff was used to identify probable MDD.

PGD was measured using the Prolonged Grief 13 (PG-13) (57). A diagnosis of PGD was determined using an algorithm that identifies a diagnosis of PGD in *DSM-V* and *ICD-11* (58). The PG-13 has been found to have high internal consistency (Cronbach’s α = 0.94) and test–retest reliability (.80) (59). The internal consistency in this sample was high (α = 0.92).

All time requirements for diagnoses were included when determining probable MHCs. To identify participants’ self-reported mental health history, participants were asked whether a doctor had previously advised that they had PTSD, MDD, an anxiety disorder, or a substance use disorder. They were then asked whether the diagnosis was “before,” “after,” or “before and after” the death. Responses to “before” and “before and after” were scored as “yes” to mental health history.

#### Satisfaction With Information and Support Provided

Participants’ satisfaction with the information provided to them following the incident was sought by asking: “how satisfied were you with the information you received from authorities about the investigation?” Satisfaction with information provided about how their relative died was obtained by asking: “to what extent have you been able to get an account of how your loved one died that you are satisfied with?” Satisfaction with support provided following the death was elicited by asking three questions: “how satisfied are you with the support you received from 1) authorities, 2) family and friends, and 3) support groups following the death?” All items used a five-point rating score (1 = very dissatisfied/not at all, 5 = very satisfied/to a large extent).

Access to counseling support was assessed by asking whether participants had used counseling services following the death (no/yes). Participants were also asked if they contacted any support groups or services following the death (no/yes) and if someone from a formal organization, support group, or support service had helped them navigate the formal processes following the death (no/yes).

### Analysis

Severity of PTSD and MDD symptoms were calculated and probable clinical conditions determined using the cutoffs identified above. Probable PGD was identified using the algorithm provided by Prigerson (58). Chi-square analysis, Fisher’s exact tests, and t-tests were used to identify bivariate relationships between the demographic, information, and support variables and each of the MHCs. Variables displaying statistically significant relationships (p < .05) with any MHC were entered into the logistic regression models. Inter-correlations between “predictor” variables were examined to check for evidence of multicollinearity and the Box-Tidwell Transformation test used to identify linearity in the logit. For ease of interpretation in the discussion of results, the odds ratios (OR) were converted to percentages [(OR-1) x 100] when less than 1.00.

#### Missing Data

Twenty cases (13.5%) had at least one missing value in the information and support variables (2.0% – 7.0%). No significant differences in demographic variables between cases with data and those without data were found. Little’s (60) MCAR test indicated that values were missing at random (χ2 = 42.65, df = 33, *p* = .121) so multiple imputation (MI) was performed to adjust for missing data (five imputations) using fully conditional specification. With the exception of gender, all variables used in analyses were included in the imputations (p. 559) (61). Analyses were conducted using the pooled results and using listwise deletion. The pattern of results was the same, so imputed values are presented. MI produces parameter estimates that are less biased than those from traditional missing data strategies, such as listwise deletion, both when data are missing at random and when data are not missing at random (62, 63). The IBM SPSS version 24 (64) was used for all statistical analyses.

## Results

### Descriptive Characteristics of Participants

The total cohort column in **Table 1** provides the descriptive characteristics of participants (n = 148). The majority were female (89.9%), reflecting evidence that approximately 90 percent of traumatic workplace fatalities are male (65). Most were partners/spouses (38.5%) or parents (35.1%) and over half (64.2%) were next of kin to the deceased worker. They were from Australia (62.2%), Canada (16.9%), the US (15.5%), and the UK (5.4%). Their mean age was 48.56 years (SD = 12.1), and the mean time since the death was 6.40 years (SD = 5.78). Most deaths occurred in the four industries that regularly account for more than 70 percent of all industrial deaths in the countries included in this study—construction, manufacturing, transport, and agriculture forestry and fishing (65).

**Table 1:** Demographic variables for total cohort (N = 148) and their associations with PTSD, MDD, and PGD.

Variable	Total cohort (N = 148)	PTSD^a^ (n = 91)	Test Statistic^b^	MDD^a^ (n = 65)	Test Statistic^b^	PGD^a^ (n = 64)	Test Statistic^b^
**Demographics**							
Females, n (%)	133 (89.86)	80 (87.91)	0.989	58 (89.23)	0.051	57 (89.06)	0.080
Age, years M (SD)	48.56 (12.13)	48.34 (12.00)	0.278	49.15 (11.42)	0.525	48.22 (11.85)	0.298
Next of kin, n (%)	95 (64.23)	62 (68.13)	1.598	49 (75.38)	6.320*	44 (68.75)	1.020
Relationship to worker, n (%)			4.549		5.876		3.445
Partner/spouse	57 (38.51)	33 (36.27)		28 (43.08)		23 (35.94)	
Parent	52 (35.13)	36 (39.56)		26 (40.00)		27 (42.19)	
Child	15 (10.15)	10 (10.98)		5 (7.69)		5 (7.81)	
Sibling	22 (14.86)	12 (13.19)		6 (9.23)		9 (14.06)	
Other^c^	2 (1.35)						
Age of worker, M (SD)	36.78 (13.43)	36.88 (13.78)	0.109	36.74 (13.69)	0.036	36.16 (14.03)	0.495
Country, n (%)			4.849		2.015		0.315
Australia	92 (62.16)	60 (65.94)		43 (66.15)		40 (62.50)	
Canada	25 (16.89)	11 (12.09)		8 (12.31)		11 (17.19)	
USA	23 (15.54)	16 (17.58)		11 (16.92)		9 (14.06)	
UK	8 (5.41)	4 (4.39)		3 (4.62)		4 (6.25)	
Mental Health History, n (%)	33 (22.29)	26 (28.57)	5.368**	21 (32.31)	6.704**	21 (32.81)	7.196**
Time since death, years M (SD)	6.40 (5.78)	4.59 (3.76)	4.569***	4.82 (4.35)	3.008**	4.31 (3.62)	4.323***
**Industry** **^d^**			6.930		8.653		9.143
Construction	55 (37.41)	28 (31.11)		18 (28.12)		18 (28.57)	
Manufacturing	24 (16.32)	17 (18.89)		13 (20.31)		13 (20.63)	
Transport	20 (13.60)	15 (16.67)		13 (20.31)		12 (19.05)	
Mining	16 (10.89)	8 (8.89)		5 (7.82)		4 (6.35)	
Agriculture/forestry/fishing	16 (10.89)	12 (13.33)		8 (12.50)		9 (14.29)	
Other	16 (10.89)	10 (11.11)		7 (10.94)		7 (11.11)	

*p < .05 **p < .01 ***p < .001, ^a^participants with probable mental health condition; ^b^chi-Square/Fishers test, t-test; ^c^other = cousins; not included in chi-square analysis, ^d^n = 147.

### Mental Health Conditions

Participants’ mean score for the PCL was 48.89 (*SD* = 17.89). Ninety-one (61.5%) scored over the diagnostic threshold score of 44, indicating probable PTSD. For the PHQ-8, the mean score was 12.05 (*SD* = 7.82), and 65 participants (44%) met the diagnostic threshold score of 10 for probable MDD. The mean severity score for the PG-13 was 37.46 (*SD* = 11.69), and 64 participants (43.2%) met the diagnostic criteria for probable PGD. Sixty-two (41.9%) participants had PTSD and MDD, 47 (31.76%) had all three MHCs, and 96 (64.9%) had any MHC. The majority (78%) did not self-report a mental health history.

### Satisfaction With Information and Support

**Table 2** provides results for participants’ satisfaction with the information and support they received following the fatality. All participants received information about the investigation and how their relative died from authorities. Sixty-nine (47%) participants received information about the investigation from two sources: the authorities and a support service. This cohort reported significantly less satisfaction with the information received from authorities (M = 2.45, SD = 1.33) than from family support groups and services (M = 3.49, SD = 1.56; *t* = −4.248, *p* < .001).

All participants reported on support from authorities. Ninety-seven (65.5%) participants received support from three sources: the authorities, a support service, and their own family network. They reported significantly less satisfaction with the support from authorities (M = 2.17, SD = 1.30), and from family and friends (M = 3.61, SD = 1.43), than from family support groups and services (M = 4.00, SD = 1.42; *t* = −12.507, *p* = .000; *t* = −2.121, *p* = .037, respectively). Relatively few participants had access to a support person following the death (n = 37, 25.0%), and about two thirds accessed counseling services (n = 102, 68.9%).

**Table 2:** Information and support variables for total cohort (N = 148) and their associations with PTSD, MDD, and PGD.

Variable	Total cohort (N = 148)	PTSD^a^ (n = 91)	Test Statistic^b^	MDD^a^ (n = 65)	Test Statistic^b^	PGD^a^ (n = 64)	Test Statistic^b^
**Information** **^c^**							
From authorities, M (SD)	2.37 (1.33)	2.17 (1.28)	2.170*	1.97 (1.12)	2.847**	2.19 (1.27)	1.173
From support service, M (SD)^d^	3.45 (1.58)	3.29 (1.69)	0.992	2.86 (1.36)	2.518*	3.32 (1.68)	0.646
How they died, M (SD)	2.86 (1.49)	2.44 (1.47)	3.843***	2.31 (1.39)	3.565***	2.29 (1.44)	3.815***
**Emotional support** **^c^**							
From authorities, M (SD)	2.05 (1.24)	1.84 (1.30)	2.248*	1.77 (1.11)	2.132*	1.98 (1.28)	0.438
From family network, M (SD)	3.60 (1.49)	3.38 (1.50)	2.425*	3.34 (1.55)	2.189*	3.28 (1.61)	2.419*
From support services, M (SD)^d^	4.00 (1.43)	4.00 (1.41)	0.168	3.76 (1.49)	1.205	3.97 (1.40)	0.017
Accessed counseling, n (%)	102 (68.9)	65 (71.43)	0.695	47 (72.31)	0.621	45 (70.31)	0.102
Contact with support group, n (%)	98 (66.22)	59 (64.84)	0.201	42 (64.62)	0.133	44 (68.75)	0.324
Had a support person, n (%)	37 (25.00)	19 (20.88)	2.140	10 (15.38)	5.715*	11 (17.19)	3.671

*p < .05 **p < .01 ***p < .001, ^a^participants with probable mental health condition; ^b^chi-Square/Fishers test, t-test; ^c^scale of 1–5, 1 = very dissatisfied/not at all, 5 = very satisfied/to a large extent; ^d^n = 69 and 95, respectively, not everyone accessed a support service, not included in further analyses.

### Bivariate Associations With Mental Health Conditions

**Tables 1** and **2** present the associations between MHCs and demographic, information, and support variables. Shorter time since the death and a mental health history were significantly related to all MHCs, as was dissatisfaction with (1) the information provided about how their relative died and (2) emotional support received from family and friends after the death. Participants with PTSD or MDD were also significantly more dissatisfied with information and support received from authorities. Elevated MDD symptoms were more likely to be reported by next of kin and those who did not have a support person following the death. There were no significant associations with country of origin (Australia, Canada, or the US) or industry in which the death occurred. Bivariate associations with comorbid mental health conditions are provided in **Tables 1** and **2** in the **Supplementary Material**

### Logistic Regression Analyses

There were no strong inter-correlations (r ≥ 0.7) identified between “predictor” variables and the Box-Tidwell Transformation test showed linearity in the logits. Variables displaying significant bivariate associations with each MHC were entered into the logistic regression model for that MHC (see **Tables 3**–**5**).

**Table 3:** Results of logistic regression models testing independent associations of key correlates with PTSD.

Key correlates	Model			
	B	SE	OR	95% CI OR
Time since death (years)	−0.184	0.045	0.832***	0.762–0.909
Mental health history^a^	−0.830	0.546	2.294	0.150–1.271
Information from authorities	−0.216	0.208	0.805	0.535–1.212
Information how died	−0.271	0.160	0.763	0.558–1.043
Support from authorities	−0.121	0.197	0.886	0.602–1.305
Support from family network	−0.331	0.164	0.718*	0.520–0.990
−2 Log Likelihood		149.24		
Nagelkerke R^2^		.378		
Classification accuracy		76.4%		
Model χ2		48.34***		

*p < .05 **p < .01 ***p < .001.

a Reference: no mental health history.

**Table 4:** Results of logistic regression models testing independent associations of key correlates with MDD.

Key correlates	Model			
	B	SE	OR	95% CI OR
Time since death (years)	−0.098	0.039	0.907*	0.840–.979
Mental health history^a^	1.134	0.508	3.107*	0.119–.871
Next of kin^b^	0.899	0.416	2.458*	0.180–.919
Information from authorities	−0.331	0.208	0.718	0.478–1.079
Information how died	−0.232	0.162	0.793	0.577–1.091
Support from authorities	−0.026	0.206	0.974	0.651–1.460
Support from family network	−0.198	0.147	0.820	0.615–1.095
Support person^c^	1.026	0.467	2.780*	1.118–6.962
−2 Log likelihood		157.80		
Nagelkerke R^2^		.352		
Classification accuracy		70.9%		
Model χ2		45.17***		

*p < .05 **p < .01 ***p < .001.

a Reference group: no mental health history;

b Reference group: not next of kin;

c Reference group: accessed support person.

**Table 5:** Results of logistic regression models testing independent associations of key correlates with PGD.

Key correlates	Model			
	B	SE	OR	95% CI OR
Time since death (years)	−0.144	0.045	0.866**	0.793–0.946
Mental health history^a^	−0.857	0.456	0.424	0.174–1.038
Information from authorities	−0.216	0.208	0.805	0.535–1.212
Information how died	−0.391	0.139	0.676**	0.515–0.888
Support from family network	−0.276	0.140	0.759	0.576–1.000
−2 Log likelihood		164.38		
Nagelkerke R^2^		.304		
Classification accuracy		67.6%		
Model χ2		38.08***		

*p < .05 **p < .01 ***p < .001.

a Reference: no mental health history.

#### Posttraumatic Stress Disorder

Of the variables included in the model, only time since death and satisfaction with emotional support provided by the family network made a significant and independent contribution to the model (see **Table 3**). The model was statistically significant and correctly classified 76.4 percent of cases. For every year of increase in the time since the death, the odds of having PTSD were reduced by a factor of 0.832 or 17 percent. Every unit increase in satisfaction with the emotional support provided by the family network lowered the risk of having PTSD by a factor of 0.718, or 28 percent.

#### Major Depressive Disorder

The MDD model with all explanatory variables was statistically significant and correctly classified 71 percent of cases (see **Table 4**). Four variables made significant independent contributions: time since death, mental health history, next of kin status, and support person. A one unit increase in time since the death reduced the odds of having MDD by a factor of.907 or 9 percent. Participants with a mental health history or next-of-kin status were 3.11 and 2.45 times more likely, respectively, to have MDD than participants who did not. Those who did not access a support person following the death were about three times more likely to have MDD than those who did.

#### Prolonged Grief Disorder

Time since the death and information about the death made a significant and independent contribution to the model (see **Table 5**). The full model was statistically significant and correctly classified 68 percent of cases. Each additional year from date of death reduced the likelihood of having PGD by a factor of 0.866 or 13 percent. Every unit increase in satisfaction with the information received about the death reduced the odds of having PGD by a factor of 0.676 or 32 percent.

## Discussion

This study documented the mental health consequences experienced by families bereaved by a sudden, fatal work injury. The findings indicate that family members across the four countries are vulnerable to high levels of probable MHCs and that these conditions are associated with various demographic, information, and support variables. The rates of MDD and PGD are comparable to those reported by family members bereaved by violent events such as homicide, suicide and accidents (17, 19–21). Comorbidity between PTSD, MDD, and PTSD was common in this cohort and echoes previous findings from studies examining bereavement from sudden and violent events (14, 66).

The rates of PTSD, however, are higher than those reported in most studies of sudden death and are more consistent with lifetime estimates following bereavement from homicide (67) and current rates following bereavement from civilian war (68). As two-thirds of the participants in this study reported having contact with a support group or service following the death, and they were, overall, dissatisfied with the information being provided by authorities, it is likely that these factors may have motivated participation. The participation by a high percentage of women may have influenced the rates of mental health conditions given the relationship between female gender and risk of developing PTSD, MDD, and PGD (32). However, as most of the victims of traumatic workplace deaths are male—96% in Australia (65), 97% in Canada (69), and 93% in the US (70)—a high proportion of next of kin and partners will be women. The high proportion of female participants in this study reflects the reality of this workplace death statistic. It is also well known that those most affected by trauma tend not to participate in research, and if they do, there is a high drop-out rate (71).

In this context, the level of PTSD experienced by participants could be viewed as somewhat more closely approximating the “real” experience of work-related traumatic bereavement for families who experience the procedural and legal formalities following the death. Their experience is one of lengthy investigations and active withholding of information by authorities due to legal privilege (24, 26). If a case is prosecuted—which is increasingly rare (72)—they witness their loved one being “barely represented” (24) or misrepresented (27) by a system in which they have no power or legal mechanisms to intervene. Tombs and Whyte (73) suggests that “the criminal justice system denies almost all occupational injury victims and bereaved relatives an admission of culpability by the guilty parties or a day in court.”

All hypothesized univariate associations between probable MHCs and the demographic, information, and support variables were significant, except for younger age in the deceased worker. These results largely support the existing trauma literature on risk factors for PTSD, MDD, and PGD (32). It is likely the lack of association between the deceased worker’s age and the MHCs partly reflects the absence of very young and very old workers in the present sample. In general, fewer fatalities occur among young workers (≤25 years) and older workers (≥ 65 years), mainly because of the lower workforce participation of these age groups in western countries (65).

Dissatisfaction with information about how the death occurred was associated with all MHCs, confirming the importance of providing accurate, timely information following the death (74). Supporting this finding, recent qualitative research has identified the value of timely and thorough coronial hearings for uncovering information about the death, underlying causal factors, the degree of pain and suffering experienced, and who or what should be held accountable for the death (75, 76). Families frequently complained about the challenges of obtaining information, reflecting similar concerns among other families dealing with sudden and violent deaths that necessitate legal involvement (74, 77–79).

The present findings suggest that greater consideration should be given to the way that families and authorities communicate, and that the ways in which the legal processes themselves affect psychological consequences for families should be closely examined [see also Refs. (79, 80)]. Timely and adequate provision of information is unlikely to be enough. Families also want prosecution of culpable parties and clear actions taken to ensure others do not experience similar tragedies. Failure to achieve either of these things can be a source of frustration, anger, and bitterness (24, 27). These findings suggest that changes are needed in policies regarding investigation and prosecution, the imposition of penalties, and remedial and preventative measures following workplace death.

Satisfaction with support from the family network was also associated with all MHCs, underlining the importance of providing adequate support following traumatic bereavement. Paradoxically, family and friends are often the least able to provide support because they may also be affected by the death, and any relationship difficulties present before the trauma may be aggravated (7, 81). It is plausible that families reach out for support from support services when support from their own network is not adequate. Satisfaction with the level of support provided by family support services was significantly higher than for support provided by authorities and the family network, possibly because support provided by people who have experienced the same type of loss is viewed as more meaningful (82, 83). The involvement of these bodies also breaks down the isolation frequently felt by families, demonstrating that their concerns and anxieties are shared. Since these bodies often engage in advocacy on behalf of families, some even building links with regulatory agencies and affecting government policies, they provide both an outlet for activism and a sense of empowerment (7, 24). The association between satisfactory support and a lower risk of developing MHCs suggests that families should be encouraged to access victim advocates, family support services, or a family liaison officer within the regulatory system to ensure support is targeted and timely (79). The victim advocacy and self-help support bodies specific to workplace death also warrant strong support to ensure the ongoing availability of the victim advocacy and peer support services needed and valued by families, given such organizations tend to have a limited life expectancy (24).

The hypothesis that participants’ satisfaction with the information and support provided following the death would be associated with a reduced risk of developing MHCs was supported for each MHC. However, the nature of the information and support that had a significant and independent association with a lower risk was different for each MHC. For PTSD, satisfaction with support from family and friends was linked with a lower risk of developing the disorder, which is consistent with existing evidence that social support acts as an important protective factor for this disorder (40, 84, 85). For MDD, access to a support person to help navigate the foreign processes and procedures following the death reduced the risk of developing the disorder; without this support, participants were nearly three times more likely to develop probable MDD. Next of kin were three times more likely to develop probable MDD. Because they are the conduit between the authorities and other family members, and often have numerous practical and legal issues to address following the death, the value of a support person who provides them with invaluable information about what to expect at each stage of the formal processes is clear [see also Ref. (79)].

Consistent with core features of PGD—difficulty accepting the loss and constant yearning for the deceased (58, 86)—participants’ satisfaction with information about the death had a significant association with a reduced the risk of developing probable PGD. However, as noted earlier in this discussion, getting information about how the death occurred in the context of a workplace death is often challenging. Information about how the death occurred is needed to process the loss (74), yet authorities (regulators and criminal justice professionals) are bound by legal processes that limit their ability to provide timely information to families even when they want to assist (26). There are similar reports of conflict between families and authorities following homicide where a lack of information creates another pathway to distress (79, 87). Reed and colleagues (79) discuss the value of having counselors or victim advocates involved in interactions with the family from the outset so that authorities can continue with their investigations while the counselor/advocate can work with the family. This approach has been adopted to some degree in relation to workplace death in Australia, with appointment of client liaison officers in some jurisdictions, but their impact has yet to be formally determined.

Although this study provides initial evidence of the mental health consequences of a sudden traumatic workplace death on surviving families, it is important to recognize that it is exploratory and has several limitations, some of which have been discussed earlier in this paper. First, the sampling may be biased because the communication channels used for recruitment may have skewed the selection of participants toward those who had access to the internet and social media, were actively seeking support, or had concerns about the information and support being provided by authorities and how investigations of the death were undertaken or reported. The survey required competence in English and therefore precluded non-English-speaking families whose culture may have shaped their bereavement process differently to the Western culture (88). A more extensive representation of families could be achieved through research partnerships with government authorities who have access to the relevant next-of-kin data that permit random sampling.

Second, the MHCs were measured by self-report questionnaire and not confirmed by clinical interviews. It may be argued that these MHCs share some features and symptoms that confound self-reporting. However, there is evidence that PTSD, MDD, and PGD are separate disorders (89–92). Additionally, the questionnaires used to measure each MHC have been widely used in screening and diagnostic studies and have high sensitivity and specificity in regard to diagnostic criteria (51, 55, 58). It should also be noted that PTSD was assessed according to the DSM-IV-TR (93) criteria rather than the criteria in DSM-V (94). The DSM-V criteria have been reported as resulting in fewer cases of PTSD mostly due to a more restrictive approach to the definition of trauma and the redefinition of avoidance (criterion C) symptoms (95, 96). It is unlikely that the changed definition of trauma (criterion A) would have resulted in fewer cases of PTSD in this study. All participants had experienced a death in the family that was, by nature, violent and unnatural therefore satisfying both DSM-IV-TR and DSM-V criteria. Less is known about the potential impact of the change in avoidance symptoms on rates of PTSD in this study because findings vary in studies that have used the two versions [for examples see Refs. (95, 97)].

Third, a relatively high proportion of those who responded to the survey provided incomplete data and were excluded from the data analysis. While demographic differences between these participants and those in final sample were identified (i.e., not immediate family, younger participants), it may be that variables that were not measured affected their participation. Finally, the research design was cross-sectional and cannot demonstrate prediction or causality. Nevertheless, in the absence of previous research on the topic, it was important to establish initial evidence that variables identified as likely predictors, or causes, of MHCs following a workplace death were at least correlated cross-sectionally before proceeding to more complex and expensive research aimed at establishing prediction or causality. In future, prospective research designs would allow for prediction and causality to be more rigorously evaluated and the best timing for tailored interventions determined.

Notwithstanding the limitations, this study had several strengths that should be noted. First, the next of kin and families of workers who have died at work are sensitive and hidden population who have rarely, if ever, been the sole focus of a mental health study. Given the frequency of fatal work incidents each year, this study fills an important gap in the literature and provides a baseline for future research. Second, there is an elaborate and specific regulatory framework that governs workplace safety and workplace deaths in most Western nations, and this study collected data from families in Australia, Canada, the US, and UK. As far as the authors are aware, it is the first study to draw on respondents from several countries, and the uniformity of the responses is striking. This suggests that the gaps and problems identified are not confined to a particular country, and the same may apply to remedial measures. Although not large, this study is a first of its kind and provides an initial appreciation of the possible similarity in mental health consequences for families from nations that have broadly similar socioeconomic and regulatory structures.

Third, the study was part of a wider project that included detailed interviews with regulatory agencies (inspectorates, workers’ compensation authorities, coroners and police) and institutions (insurers, unions, employers, and lawyers) who deal with families following a workplace death. Their views provided a detailed understanding of how institutions respond to sudden work deaths, including the provision of information, counseling, financial, and other supports (26, 98). For example, the families of self-employed workers—a growing segment of the workforce in the gig economy—are largely denied access to workers’ compensation, and this financial burden causes considerable concern and financial distress, possibly exacerbating the mental anguish that families experience (28). Taken with findings from other parts of this study, the present findings reinforce the need for changes to regulatory policies, and financial and other supports, afforded to bereaved families.

In conclusion, this study produced promising initial evidence that families who experience a traumatic workplace death are at increased risk of developing PTSD, MDD, and PGD, and that several information and support variables are associated with increased or decreased risk of developing these conditions. Satisfaction with family support was a protective factor for PTSD and satisfactory information about the death reduced the likelihood of PGD. Next of kin were at increased risk of having MDD, and having a support person to help navigate the legal and procedural formalities following the death reduced this risk. The findings reinforce previous research pointing to the importance of more timely and adequate provision of information to families, as well as the need for effectiveness in types of family support services and support people. However, further research is needed to build on these broad initial findings. Research questions that focus on the specific formalities and processes following the death would provide more detailed evidence of the benefits and challenges that families and authorities encounter and more fully inform clinicians about how it is best to design interventions to mitigate the mental health impact of workplace death on families.

## Data Availability

The datasets generated for this study will not be made publicly available. The study protocol approved by the Human Research Ethics Committee does not include the sharing or reuse of data.

## Author Contributions

All authors conceived and designed the study and were investigators on the supporting grant. LM analyzed the data and drafted the initial manuscript. All authors contributed to the manuscript revision and read and approved of the final draft of the manuscript before submission.

## Funding

This research was supported by a grant from the Australian Research Council (DP120103377).

## Conflict of Interest Statement

MQ was a patron of the Workplace Tragedy Family Support Group until it ceased in 2019.

The remaining authors declare that the research was conducted in the absence of any commercial or financial relationships that could be construed as a potential conflict of interest.
