# Supplementary material for: Posttraumatic Stress Disorder, Depression, and Prolonged Grief Disorder in Families Bereaved by a Traumatic Workplace Death: The Need for Satisfactory Information and Support
Source: Front Psychiatry. 2019 Aug 30;10:609. doi: 10.3389/fpsyt.2019.00609 (PMC6728923; doi:10.3389/fpsyt.2019.00609)
Supplement: Supplementary file 1 [file DataSheet_1.pdf]

Supplementary information

Supplementary Table 1. Demographic variables for total cohort (N = 148) and their associations with comorbid conditions

Variable	Total cohort (N = 148)	PTSD/MDD[a] (n = 62)	Test Statistic[b]	PTSD/PGD[a] (n = 60)	Test Statistic[b]	MDD/PGD[a] (n = 49)	Test Statistic[b]	All MHCs (n = 47)	Test statistic [b]
Demographics									
Females, n (%)	133 (89.86)	55 (88.71)	0.156	53 (88.33)	0.260	45 (91.84)	0.313	43 (91.49)	0.200
Age, years M (SD)	48.56 (12.13)	49.08 (11.68)	-0.441	48.45 (11.94)	0.091	48.20 (11.55)	0.251	48.17 (11.80)	0.266
Next-of-kin, n (%)	95 (64.23)	47 (75.81)	**6.265***	42 (70.0)	1.482	37 (75.51)	**4.084***	36 (76.59)	**4.612***
Relationship to worker, n (%)			3.609		1.691		3.587		2.943
Partner/spouse	57 (38.51)	26 (41.93)		22 (36.67)		21 (42.86)		20 (42.55)	
Parent	52 (35.13)	25 (40.32)		25 (41.67)		20 (40.82)		19 (40.43)	
Child	15 (10.15)	5 (8.08)		5 (8.33)		4 (8.16)		4 (8.51)	
Sibling	22 (14.86)	6 (9.67)		8 (13.33)		4 (8.16)		4 (8.51)	
Other[c]	2 (1.35)								
Age of worker, M (SD)	36.78 (13.43)	36.71 (13.78)	0.409	36.48 (14.04)	0.224	36.80 (14.31)	0.008	36.77 (14.30)	0.011
Country, n (%)			2.648		0.481		0.712		0.785
Australia	92 (62.16)	41 (66.13)		39 (65.00)		32 (65.31)		31 (65.96)	
Canada	25 (16.89)	7 (11.29)		10 (16.67)		8 (16.33)		7 (14.89)	
USA	23 (15.54)	11 (17.78)		8 (13.33)		6 (12.24)		6 (12.76)	
UK	8 (5.41)	3 (4.80)		3 (5.00)		3 (6.12)		3 (6.39)	
Mental Health History, n (%)	33 (22.29)	20 (32.25)	**6.110***	20 (33.31)	**7.094****	17 (34.69)	**6.497***	16 (34.04)	**5.484***

Time since death, years M (SD)	6.40 (5.78)	4.68 (4.12)	**3.382****	4.45 (3.68)	**3.876*****	4.60 (3.86)	**3.188****	4.67 (3.91)	**2.971****
Industry[d]			10.109		8.425		9.906		
Construction	55 (37.41)	17 (27.87)		17 (28.81)		13 (27.08)		12 (26.09)	9.897
Manufacturing	24 (16.32)	12 (19.67)		12 (20.33)		11 (22.92)		10 (21.74)	
Transport	20 (13.60)	13 (21.31)		12 (20.33)		11 (22.92)		11 (23.91)	
Mining	16 (10.89)	4 (6.56)		4 (6.78)		3 (6.24)		3 (6.52)	
Agriculture/forestry/fishing	16 (10.89)	8 (13.11)		8 (13.60)		5 (10.42)		5 (10.87)	
Other	16 (10.89)	7 (11.48)		6 (10.15)		5 (10.42)		5 (10.87)	

Notes: * p < .05 ** p < .01 *** p < .001, a. participants with probable comorbidities; b. Chi-Square/Fishers test, t-test c. other = cousins; not included in chi-square analysis, d. n = 147.

Supplementary Table 2. Information and support variables for total cohort (N = 148) and their associations with comorbid conditions

Variable	Total cohort (N = 148)	PTSD/MDD[a] (n = 62)	Test Statistic[b]	PTSD/PGD[a] (n = 60)	Test Statistic[b]	MDD/PGD[a] (n = 49)	Test Statistic[b]	All MHCs (n= 47)	Test statistic[b]
**Information**[c]									
From authorities, M (SD)	2.37 (1.33)	2.92 (1.09)	**2.906****	2.21 (1.28)	1.223	1.98 (1.13)	**2.472***	1.96 (1.18)	**2.567***
From support service, M (SD)[d]	3.45 (1.58)	2.81 (1.71)	**2.663****	3.25 (1.70)	0.970	2.83 (1.71)	**2.309***	2.78(1.73)	**2.400***
How they died, M (SD)	2.86 (1.49)	2.23 (1.36)	**3.998*****	2.89 (1.46)	**3.575*****	2.18 (1.37)	**3.703*****	2.14 (1.39)	**3.801*****
**Emotional support**[c]									
From authorities, M (SD)	2.05 (1.24)	1.69 (1.04)	**2.823****	1.91 (1.76)	1.076	1.85 (1.19)	1.186	1.75 (1.11)	1.751
From family network, M (SD)	3.60 (1.49)	3.26 (1.55)	**2.456***	3.22 (1.71)	**2.635****	3.16 (1.65)	**2.452***	3.08 (1.64)	**2.806****
From support services, M (SD)[d]	4.00 (1.43)	3.70 (1.51)	1.489	3.89 (1.45)	0.467	3.67 (1.52)	1.456	3.57 (1.53)	1.819
Accessed counselling, n (%)	102 (68.9)	45 (72.58)	0.668	43 (71.67)	0.356	37 (75.51)	1.486	36 (76.59)	1.895
Contact with support group, n (%)	98 (66.22)	40 (64.52)	0.138	40 (66.67)	0.009	32 (65.31)	0.027	30 (63.83)	0.138
Had a support person, n (%)	37 (25.00)	9 (14.52)	**6.255***	10 (6.67)	3.737	8 (16.32)	2.939	7 (14.89)	3.752

Notes: * p < .05 ** p < .01 *** p < .001, a. participants with probable comorbid mental health condition; b. Chi-Square test, t-test; c. scale of 1 – 5, 1 = very dissatisfied/not at all, 5 = very satisfied/to a large extent d. n = 69 and 95, respectively, not everyone accessed a support service.
